# Temporal Control of the Host–Guest Properties of a Calix[6]arene Receptor by the Use of a Chemical Fuel

**DOI:** 10.1021/acs.joc.2c00050

**Published:** 2022-02-23

**Authors:** Francesco Rispoli, Emanuele Spatola, Daniele Del Giudice, Roberta Cacciapaglia, Alessandro Casnati, Laura Baldini, Stefano Di Stefano

**Affiliations:** † Dipartimento di Scienze Chimiche, della Vita e della Sostenibilità Ambientale, Università degli Studi di Parma, Parco Area delle Scienze 17/A, Parma 43124, Italy; ‡ Dipartimento di Chimica, Università di Roma La Sapienza and ISB-CNR Sede Secondaria di Roma - Meccanismi di Reazione, P.le A. Moro 5, Roma I-00185, Italy

## Abstract

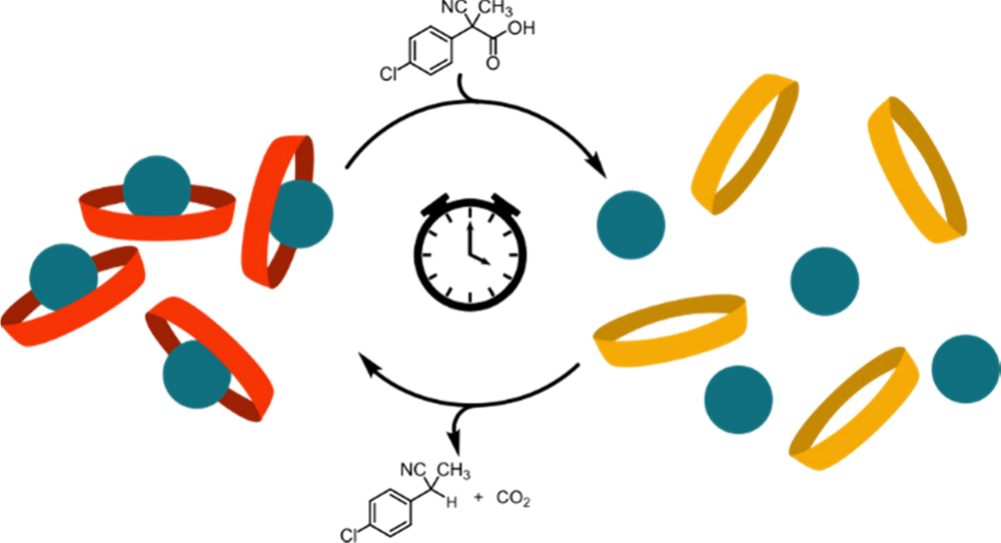

The host–guest interaction of a 1,3,5-trisaminocalix[6]arene receptor with *N*-methylisoquinolinium trifluoromethanesulfonate (*K*_ass_ of 500 ± 30 M^–1^ in CD_2_Cl_2_) can be dissipatively driven by means of 2-cyano-2-(4′-chloro)phenylpropanoic acid used as a convenient chemical fuel. When the fuel is added to a dichloromethane solution containing the above complex, the host is induced to immediately release the guest in the bulk solution. Consumption of the fuel allows the guest to be re-uptaken by the host. The operation can be satisfactorily reiterated with four subsequent additions of fuel, producing four successive release–reuptake cycles. The percentage of the guest temporarily released in the bulk solution by the host and the time required for the reuptake process can be finely regulated by varying the quantities of added fuel.

## Introduction

The capability of maintaining a functional state under the action of a chemical or photochemical stimulus is probably the characterizing feature of any chemical system with life-like properties.^1^ For this reason, a great effort is nowadays devoted to the design of artificial machineries that operate under dissipative conditions, that is until the stimulus (fuel) is present.^1a,2^ A number of dissipative systems have been recently developed in the fields of self-assembly,^3^ DNA-based systems,^4^ molecular machines and pumps,^1a,2,5^ and host–guest chemistry,^6^ with a clear predominance of the first category. In the case of dissipative host–guest chemistry, in 2015, Nitschke et al.^6a^ described the release–reuptake of a fullerene guest from and to a metal–organic architecture that disassembled and reassembled under the action of a chemical fuel (thus, in this case, the identity of the host is lost during the release phase); in 2021, we reported^6b^ on the temporal control of alpha-cyclodextrin–*para*-aminobenzoic acid interactions driven by programmed pH variations enabled by the consumption of the nitroacetic acid fuel, and Schmittel et al. described a system in which a secondary ammonium/amino axis is dissipatively hosted inside the cavity of a crown-ether derivative.^7^

**Chart 1:**
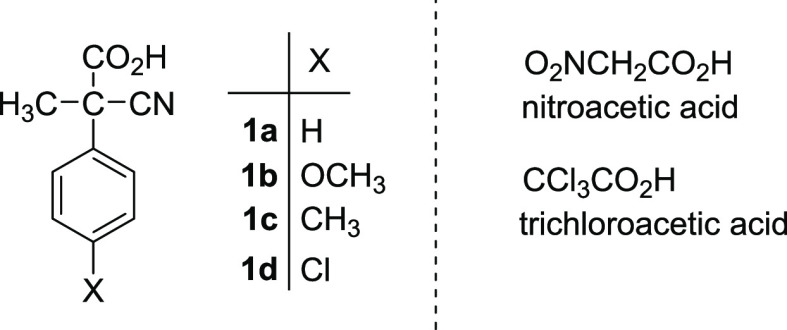
Activated carboxylic acids employed for the operation of molecular machines.

**Figure 1:**
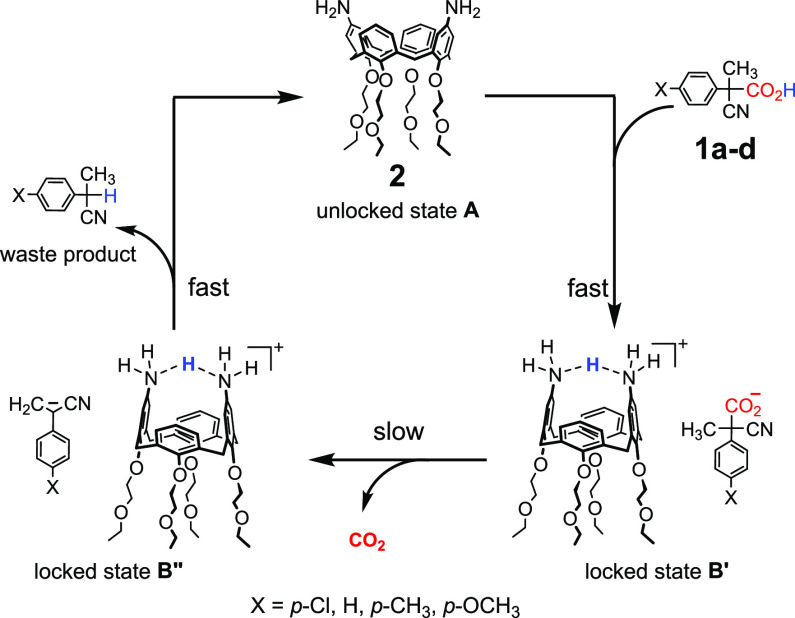
Locking–unlocking of the calix[4]arene scaffold of compound **2**, due to the action of the fuel acid, which loses CO_2_ during the operation. The system operates in dichloromethane.

In the last years, activated carboxylic acids like 2-cyano-2-phenylpropanoic acid^8^ (**1a**) and its derivatives (**1b–d**),^9^ trichloroacetic acid,^10^ and nitroacetic acid^4i^ (Chart 1) have been conveniently used as chemical fuels to drive whole cycles of motion of molecular machines, both switches and motors. Very recently, we reported that the calix[4]arene scaffold of **2** (Figure 1) can be reversibly locked and unlocked in a time-controlled fashion by means of acids **1a**–**d** used as chemical fuels.^9c^

The two amino groups implanted on the opposite positions of the upper rim of calix[4]arene **2** allow the locking/unlocking motion. It was demonstrated that such −NH_2_ groups share the proton received by the fuel acid, with a consequent locking of the structure (locked states **B′** and **B″**, see Figure 1). The locked shape is maintained until the decarboxylation of the fuel acid is complete (fuel exhaustion). At this point, the calix[4]arene is found again in its unlocked form **A** (Figure 1).

Most importantly, it was demonstrated that the duration of the locking/unlocking conformational cycle can be controlled by varying the nature of the fuel acid (increasing times in the series X = Cl, H, CH_3_, and OCH_3_) or the amount of added fuel (the greater the excess of added fuel with respect to the calix[4]arene, the longer the duration of the conformational cycle).^9c^ Subsequently, such study has found application in the temporal control of the fluorescence properties of a similar diaminocalix[4]arene further endowed with two pyrenyl moieties on the remaining opposite positions of the upper rim.^10b^

**Figure 2:**
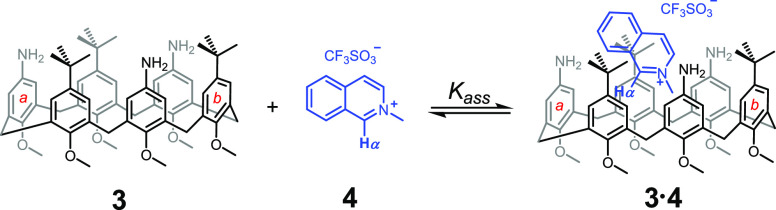
Host–guest interaction between calix[6]arene **3** and *N*-methylisoquinolinium trifluoromethanesulfonate (**4**).

We now resort to the calix[6]arene **3** (see Figure 2) where three alternate aromatic moieties are functionalized with three amine groups. Although floppier and less organized than the calix[4]arene scaffold, the calix[6]arene cavity is larger and potentially capable of hosting guest molecules.^11^ Our purpose is to drive in a temporally controlled fashion the host–guest interactions between host **3** and a suitable guest molecule by means of carboxylic acid fuels under dissipative conditions. Time control of the protonation state of the amino groups of **3** should allow the dissipative release–reuptake of the guest.

## Results and Discussion

### Synthesis

Synthesis of compound **3**(12) was attained in four steps from 1,3,5-trimethoxycalix[6]arene^13^ following a literature procedure.^12^ Compound **4**(14) was prepared as previously described.

### Binding and Base Properties of **3**

**Figure 3:**
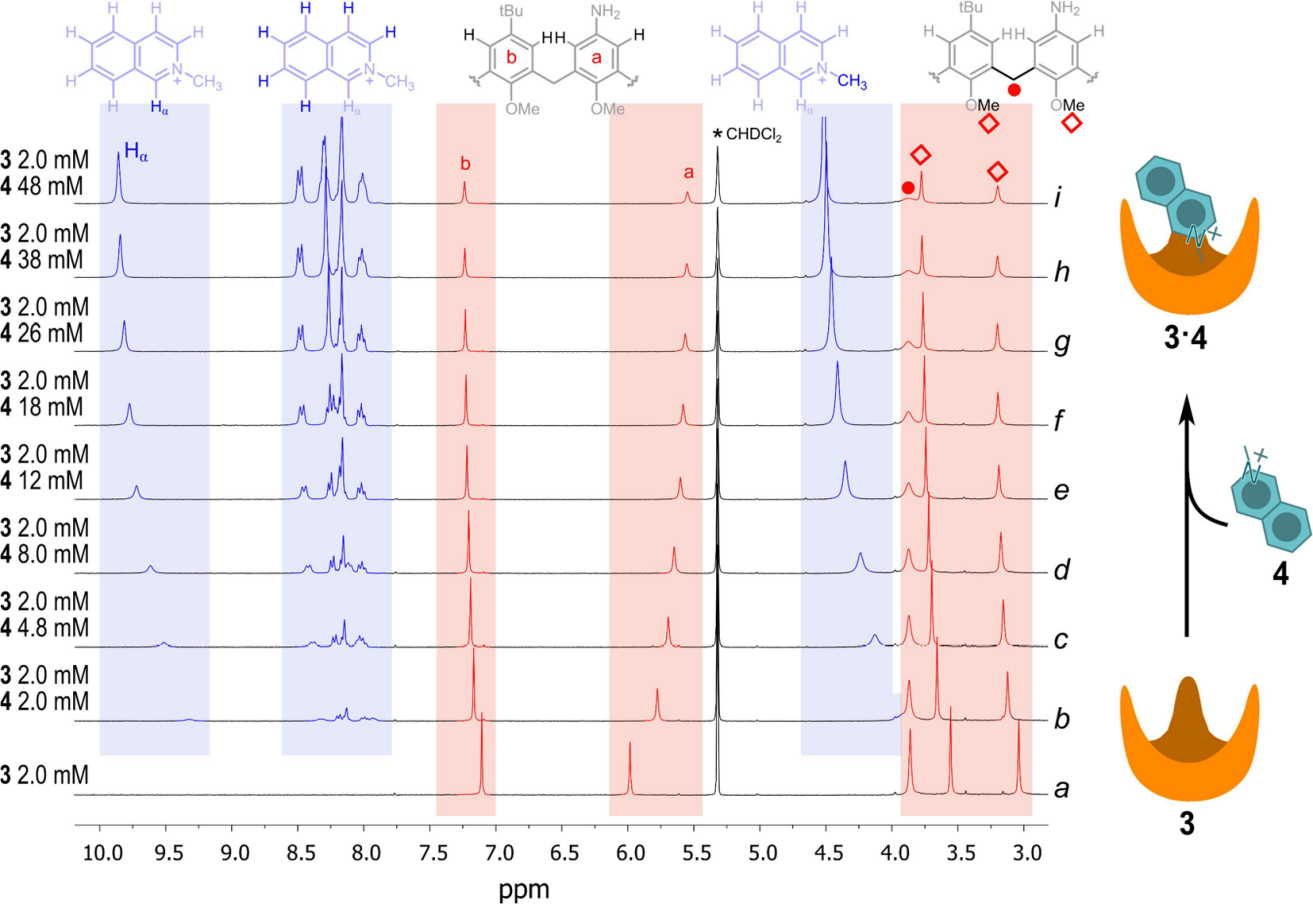
Titration of 2.00 mM calix[6]arene **3** (light red) with **4** (light blue), in CD_2_Cl_2_ at 25 °C (see Figure S2 for the complete spectra). Trace *a* is related to 2.00 mM calix[6]arene **3**; traces *b*–*i* are related to the same solution to which the given concentration of guest **4** was added (concentration of **3** was maintained constant along the titration).

A significant portion of the ^1^H-NMR (300 MHz) spectrum in CD_2_Cl_2_ at 25 °C of **3** is reported as the trace *a* of Figure 3 (see bottom trace in Figure S2, in the Supporting Information for the entire spectrum). The two singlets at 3.0 and 3.6 ppm correspond to the methoxy groups, while the singlet at 3.8 ppm is due to the 12 protons of the methylene bridges. Singlets at 6.0 ppm and 7.2 ppm are due to the aromatic protons of rings *a* and *b*, respectively.

The presence of a single singlet for the protons of the methylenic bridges indicates that calix[6]arene **3** is conformationally flexible,^15^ due to a fast rotation of the aromatic rings around the methylene hinges on the NMR time-scale. With the aim at testing *N*-methylisoquinolinium trifluoromethanesulfonate **4** as a suitable guest for calix[6]arene **3**, the latter was titrated with **4** in CD_2_Cl_2_ by ^1^H NMR. The resulting spectra are reported in Figure 3. A saturation behavior is apparent following the signals related to host **3**. The aromatic singlets of moieties *a* and *b* diverge on increasing concentrations of **4**. In the meantime, the methoxy signals are down-field shifted, and the singlet related to the methylene bridges becomes broader and broader, due to a rigidification of the structure when guest **4** is hosted inside **3**. This loss of conformational flexibility is confirmed by the low-temperature spectra of **3** in presence of a 50-fold molar excess of **4**, with the appearance, at −10 °C, of two doublets for the methylene protons (see SI, Figure S5), typical of a blocked cone conformation of the calixarene.^16^ At the same temperature, in the absence of the guest, the methylene protons give a single, slightly broadened signal (see SI, Figure S4). The fit of the experimental points obtained in the titration of **3** with **4** with a 1:1 binding isotherm gives a binding constant *K*_ass_ of 500 ± 30 M^–1^ with a very nice accordance between the calculated curve and the experimental data (see Figure S3).

Interestingly, along the titration of **3** with **4**, a significant shift of the signals of the guest **4** is also observed. In the first point of the titration (see Figure 3, trace *b*), when added titrant **4** is equimolar to calix[6]arene host **3**, the percentage of added **4** complexed to **3** is significant (38%). Inclusion of guest **4** into the aromatic cavity of host **3** is proved by the marked shielding effect observed on the signals of the guest upon complexation. Under the above conditions, a 0.68 ppm up-field shift is observed on the signal of the methyl group of the guest (δ = 4.61 ppm is the chemical shift of the methyl group of uncomplexed **4**, and δ = 3.93 ppm is the observed chemical shift in trace *b*). Among the aromatic signals related to **4**, that of the alpha proton (**H**_α_) is the most up-field shifted (it is found at 9.30 ppm in trace *b* of Figure 3 as a broad singlet and at 9.95 ppm in uncomplexed **4** as a sharper singlet) indicating that the inclusion must occur as depicted in Figure 2.

**Figure 4:**
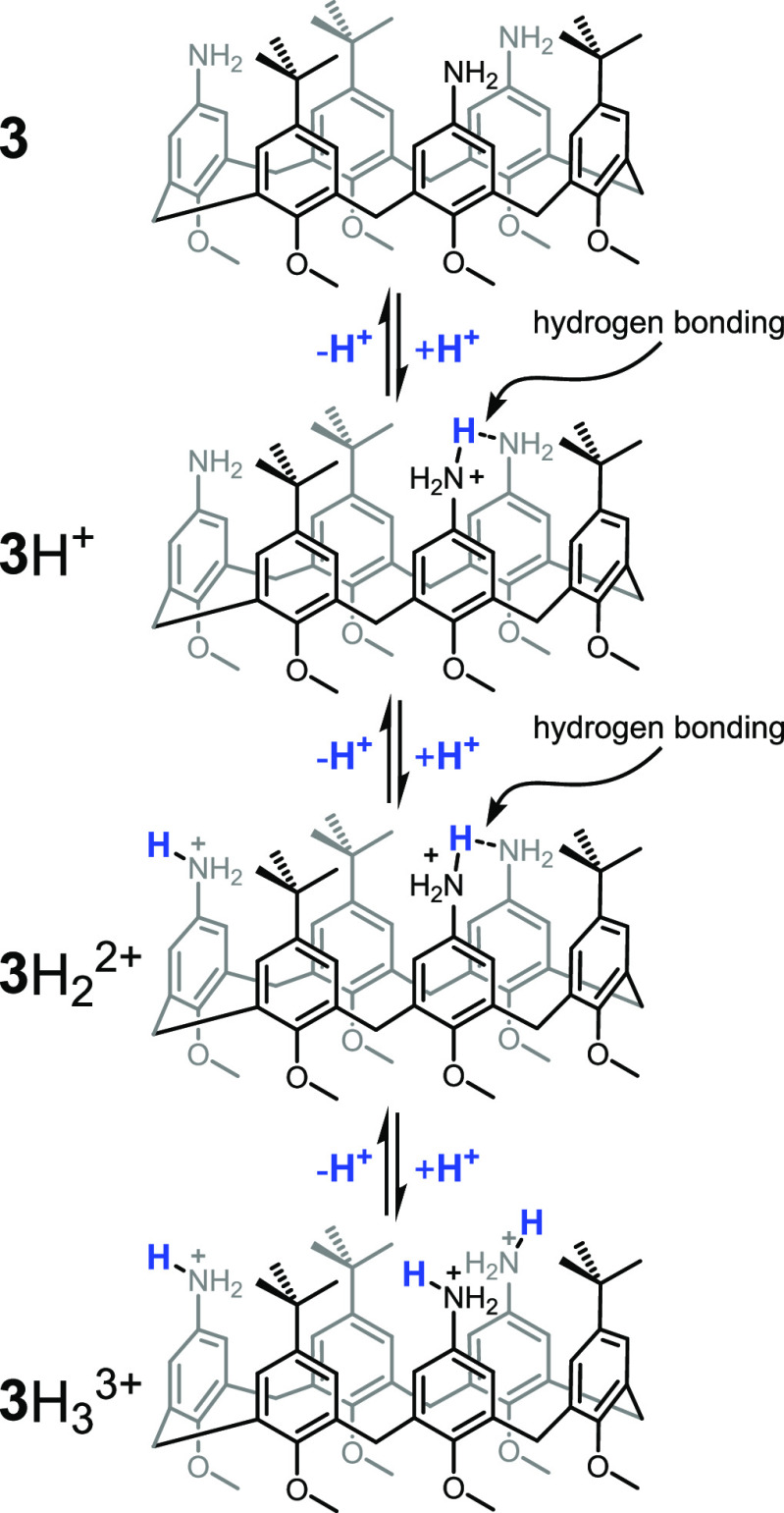
Protonation equilibria involving calix[6]arene **3** in excess of TFA.

Next, we tested the basic properties of calix[6]arene **3** by titration with trifluoroacetic acid (TFA). Addition of the first molar equiv of TFA to a 2.00 mM solution of **3** causes a general down-field shift of all signals due to the protonation of one of the three amino groups (see Figure S6). Further significant variations are observed when a second and a third molar equiv of TFA are added to the solution. In the latter case, most of the signals related to **3** appear broader, and some of them begin to show multiplicity as a consequence of an increased conformational rigidity. ^1^H NMR spectra (see Figures S6 and S7) do not seem to change appreciably upon subsequent additions of TFA (up to 12 molar equiv). A definite assessment of the protonation state of **3** after the addition of 3 or more molar equiv of TFA is not easy. Doubly protonated **3**H_2_^2+^ with one of the two protons on one of the three amino groups and the other shared between the remaining two amino groups (see Figure 4), or, alternatively, a triply protonated form (**3**H_3_^3+^) with one proton on each of the amino groups, could be formed. However, the doubly protonated state **3**H_2_^2+^ seems to be more probable taking into account the following arguments. First, in strict analogy to what was observed in the case of the monoprotonated form of calix[4]arene **2**H^+^ (see Figure 1),^9c^ the monoprotonated form **3**H^+^ obtained by the addition of one molar equiv of TFA to **3** is not deprotonated in any extent by the addition of excess *p*-anisidine up to 20 molar equiv (see Figure S8), proving that **3** has a much more basic character than *p*-anisidine probably due to the chance given to two amino groups to share the proton^17^ (see Figure 4). Second, and more importantly, calix[6]arene **3**, differently from *p*-anisidine, is basic enough to promote the decarboxylation of fuel **1d** (*vide infra*). An additional clue in favor of the **3**H_2_^2+^ hypothesis in the presence of 3 or more molar equiv of TFA, comes from the pattern of the methylene bridge signals of the ^1^H NMR spectrum obtained under these conditions (see Figure S6). They appear as two broad doublets, a typical feature of an almost blocked or slowly interconverting cone conformation. Such conformation is hardly ascribable to a triprotonated **3**H_3_^3+^ structure, which would adjust to keep the three positive groups as far as possible but is fully compatible with the **3**H_2_^2+^ form in which the two protons can be rapidly scrambled among the three amino groups.

**Figure 5:**
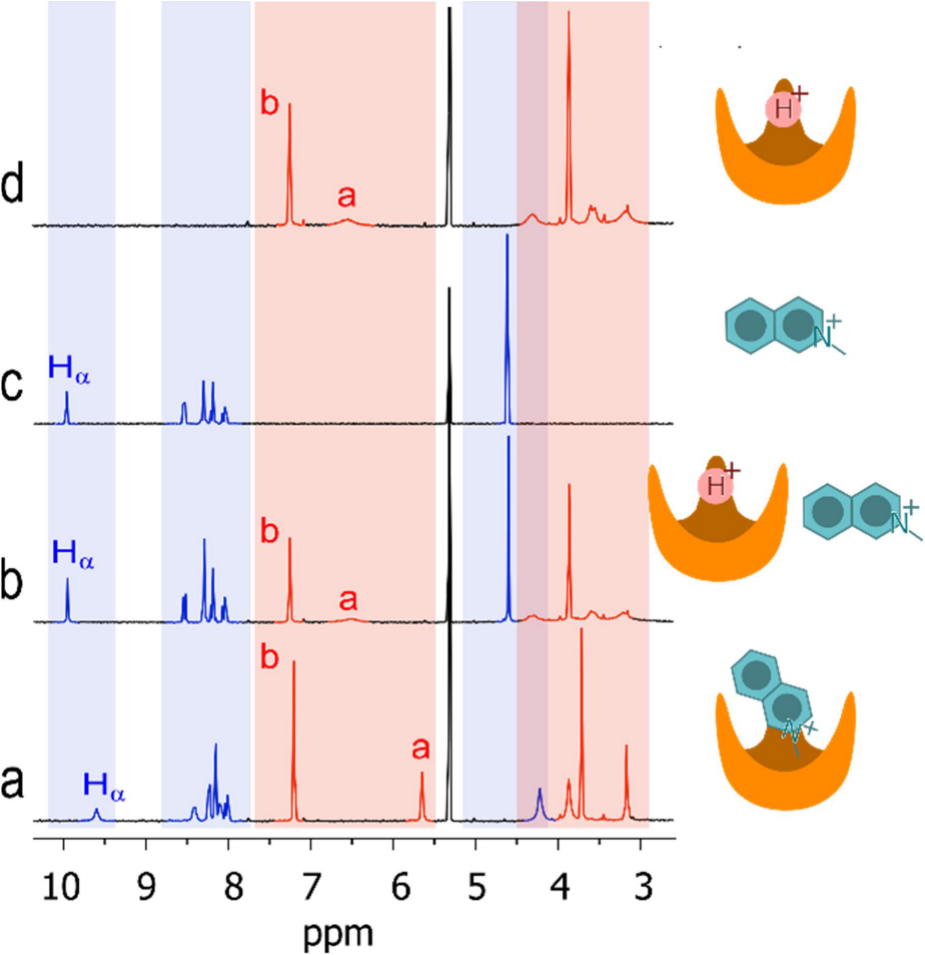
Effect of protonation on the binding ability of **3** (light red) toward *N*-methylisoquinolinium triflate **4** (light blue). (a) 2.00 mM **3** and 6.00 mM **4**; (b) 2.00 mM **3** and 6.00 mM **4** and 6.00 mM TFA; (c) 6.00 mM **4**; (d) 2.00 mM **3** and 6.00 mM TFA. CD_2_Cl_2_, 25 °C (see Figure S9 for complete spectra). Signal labeling as in Figure 3.

Figure 5 shows the effect of protonation on the binding ability of **3** toward *N*-methylisoquinolinium triflate **4**.

Trace *a* is the ^1^H NMR spectrum of a CD_2_Cl_2_ solution of 2.00 mM **3** and 6.00 mM **4**. Under these conditions, 70% **3** is in the form of **3**•**4** complex. The addition of 3 molar equiv of TFA causes the complete expulsion of guest **4** from host **3** as can be seen in the resulting trace *b* of Figure 5. Trace *b* is indeed the sum of the ^1^H NMR spectrum of **4**, trace *c*, and that of **3** + 3 molar equiv of TFA, trace *d*. The reason is probably electrostatic in nature: once host **3** is protonated, it loses any affinity for the positively charged *N*-methylisoquinolinium.

### Operation of Receptor **3** under Dissipative Conditions

**Figure 6:**
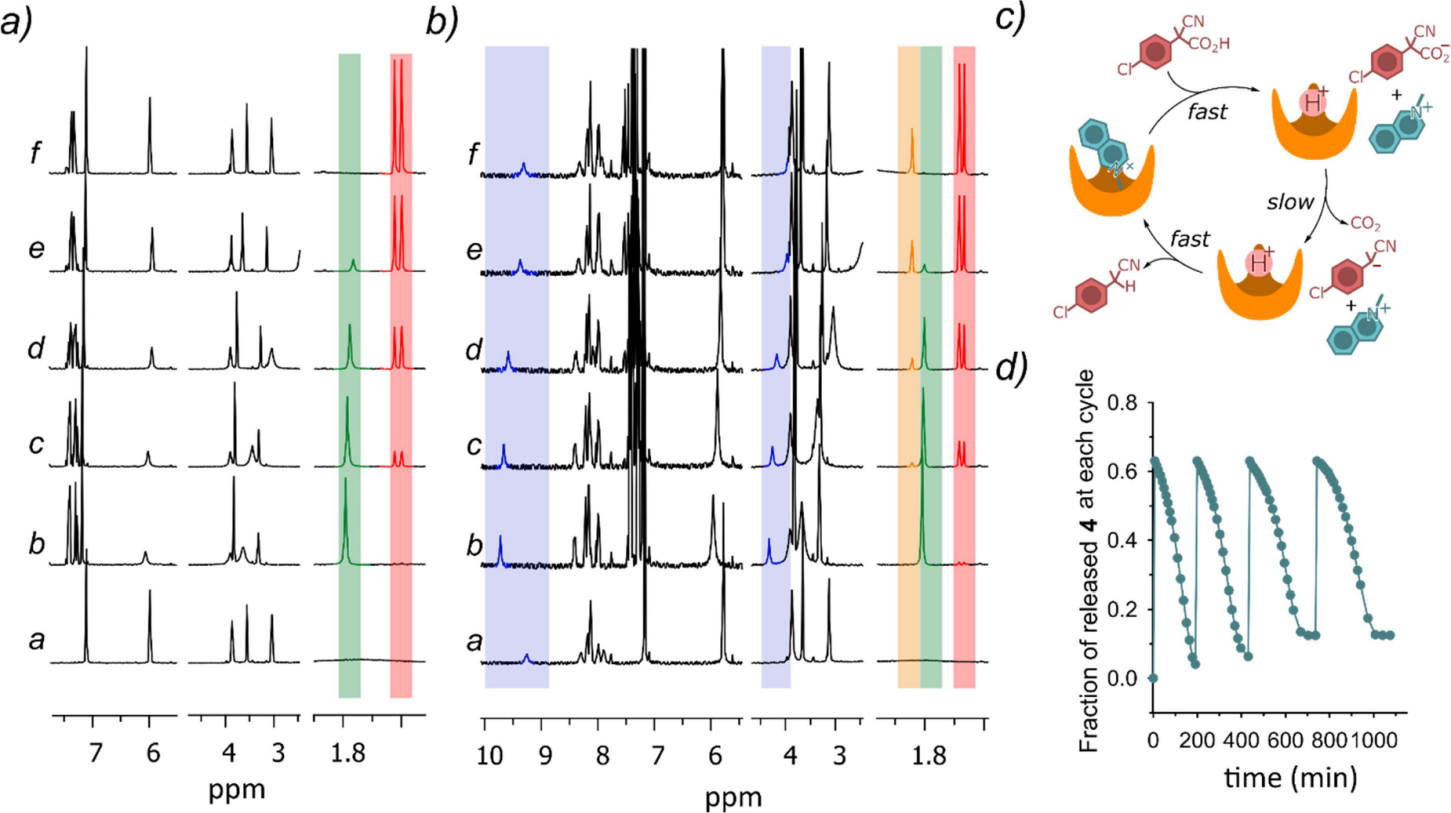
(a) Reaction between 2.00 mM **3** and 6.00 mM **1d** in CD_2_Cl_2_ at 25 °C followed by ^1^H NMR (300 MHz); trace *a* was recorded before the addition of **1d**, traces *b*, *c*, *d*, *e*, and *f* were recorded from 6 to 370 min. (b) Reaction between 2.00 mM **3** and 6.00 mM **1d** in the presence of 2.00 mM **4** in CD_2_Cl_2_ at 25 °C followed by ^1^H NMR (300 MHz); trace *a* was recorded before the addition of **1d**, traces *b*, *c*, *d*, *e*, and *f* were recorded from 6 to 177 min. (c) Schematic cartoon representing the release–reuptake **3•4** → **3**H^+^ + **4 → 3•4** dissipative cycle driven by fuel **1d**. (d) Four subsequent **3•4** → **3**H^+^ + **4 → 3•4** dissipative cycles triggered by four successive additions of fuel **1d** (in each cycle, 3 mol equiv of **1d** were added to equimolar 2.00 mM of **3** and **4**).

First of all, we investigated if calix[6]arene **3** is able to promote the decarboxylation of the fuel acids. As expected, it was found that the decarboxylation of acid **1d** (the most activated among 2-cyano-2-phenylpropanoic acids) promoted by **3** occurs in relatively quick times (the reaction is complete within 6.2 h in CD_2_Cl_2_ at 25 °C).^18^ To an initial 2.00 mM **3** solution (trace *a*, Figure 6a), fuel **1d** (3 molar equiv) is added and **3** is immediately protonated (trace *b*, Figure 6a). Decarboxylation starts and, within 6.2 h, the singlet at 1.75 ppm due to the methyl group of the deprotonated form of **1d**, is transformed into the doublet at 1.60 ppm belonging to the waste product. The typical quartet at 3.95 ppm of the benzylic proton of the waste product also appears (see Figure S10 for a better view).

Contextually, calix[6]arene **3** returns to its original nonprotonated form. Interestingly, the reaction does not occur to any extent within the same time when *p*-anisidine (6.00 mM) is added instead of **3**, strongly pointing to the sharing of the proton between two of the three amino groups in **3**H^+^ as a reason for the increased basicity of **3**. The same experiment was then repeated in the presence of 2.00 mM **4**.

**Figure 7:**
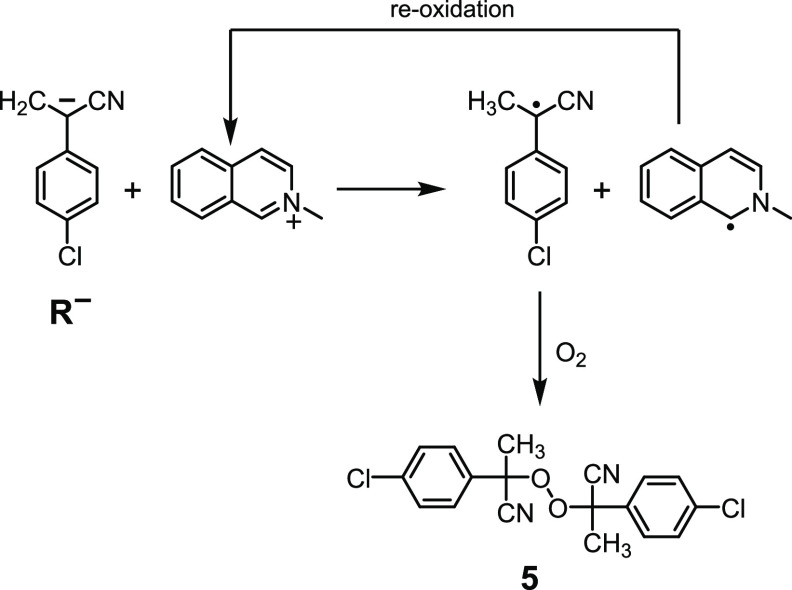
Proposed mechanistic pathway to peroxide **5**.

Under these conditions, 38% **3** is engaged in the formation of complex **3**•**4** (trace *a*, Figure 6b). Now, fuel **1d** is added (3 molar equiv with respect to **3**), host **3** is immediately protonated, and guest **4** is released into the bulk solution (trace *b*, Figure 6b). Decarboxylation of the deprotonated form of **1d** slowly occurs, **3** is contextually deprotonated again, and **4** is re-uptaken by host **3** (traces *c*–*f*, Figure 6b). Thus, a release-and-reuptake cycle of guest **4** from and into host **3** has been realized under dissipative conditions (Figure 6c).^19^ The process can be satisfactorily reiterated as shown by Figure 6d, where four subsequent cycles triggered by four successive additions of fuel **1d** are monitored by following the variation of the chemical shift of the methyl group of **4** over the time. Unexpectedly, during this release–reuptake experiment (Figure 6b), a second waste product accounting for about 25% of **1d** (Figure 6b, trace *f*, yellow singlet at 1.95 ppm to be compared with the red doublet at 1.60 ppm) appears among the reaction products. Peroxide **5** in Figure 7 was tentatively proposed as this unexpected waste product (see SI, pages S14–S15). It does not form in the absence of **4** (Figure 6a). Furthermore, such a product is not seen when the same reaction is carried out in the presence of **4** but substituting **3** with Et_3_N (a base able to promote the decarboxylation of **1d**). In other words, it is only formed when calix[6]arene **3** and *N*-methylmethylisoquinolium triflate are both present in the reaction mixture. We hypothesize that it is due to the oxidation of the carbanion by **4**,^9b,20^ immediately followed by oxygen capture, see Figure 7. The resulting *N*-methylisoquinoline radical must be then re-oxidized to *N*-methylisoquinolinium (very likely by O_2_) since the latter is found untouched at the end of each cycle.

Thus, this collateral pathway, which is somewhat facilitated by calix[6]arene **3**,^21^ does not interfere on the release–reuptake cycles. Interestingly, the contribution of the collateral pathway definitely decreases (passing from 25 to 15%) when the exclusion of O_2_ from the solution was attempted through freeze–pump–thaw cycles operated on the NMR tube or when higher excesses of fuel **1d**, 10.5 mM (5.25 mol equiv) and 18.4 mM (9.2 mol equiv) were added (passing from 25 to 15 and 10%, respectively), *vide infra*.

**Figure 8:**
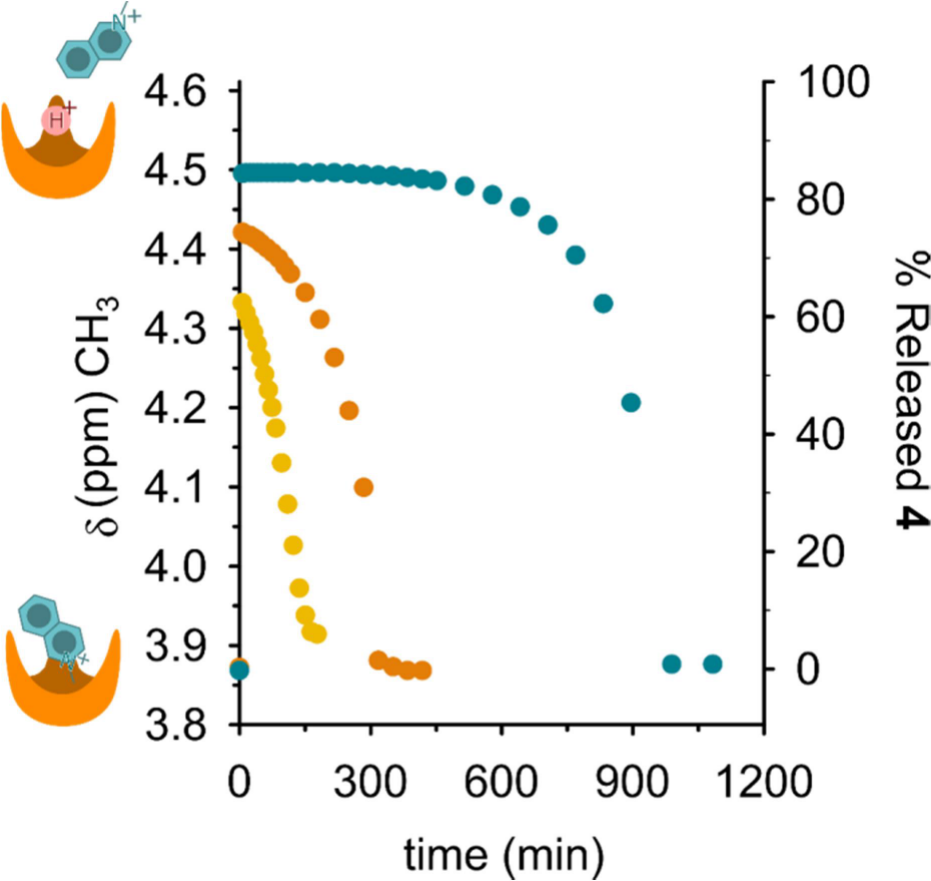
Time-controlled dissipative **3•4 → 3**H^+^ + **4 → 3•4** cycles triggered by increasing amounts of fuel **1d** (CD_2_Cl_2_, 25 °C). In all experiments, equimolar amounts of **3** and **4** were used (2.0 mM) while concentration of **1d** was varied as follows: (1) 6.0 mM (yellow), (2) 10.5 mM (orange) and (3) 18.2 mM (azure). The advancement degree of the reaction over time (right axis) was calculated as [(δ – 3.87)/(4.61–3.87)], where δ is the chemical shift of the *N*-methylisoquinolium methyl signal (left axis) observed at a given reaction time, 4.61 is the chemical shift of the methyl signal of uncomplexed *N*-methylisoquinolium, and 3.87 is the chemical shift of the *N*-methylisoquinolium methyl signal under the initial conditions, that is, when 38% complex **3**•**4** formation is observed (which corresponds to 0.76 mM **3**•**4**).

Eventually, time control of the dissipative release–reuptake cycle could be achieved by varying the amount of fuel **1d** added in the solution. Figure 8 shows three experiments where increasing amounts of fuel **1d** were added to mixtures of **3** (2.0 mM) and **4** (2.0 mM). The percentage of **4** temporarily escaped from **3** and the time spent outside increase on increasing the amount of the fuel added. When 3 molar equiv of **1d** are added (6.0 mM, Figure 8, yellow points), 63% of the guest is released by the host, and the reuptake process takes 150 min, when 5.25 molar equiv of **1d** are added (10.5 mM, Figure 8, orange points), 75% of **4** is released and reuptake takes 300 min, eventually, when 9.2 molar equiv of **1d** are added (18.4 mM, Figure 8, azure points), 85% of **4** is released and reuptake takes more than 900 min. In other words, the higher the fuel excess, the longer the time needed to consume such excess, the longer the time spent by the host in the unloaded state. The particular shape of the kinetic profiles in Figure 8 (see also Figures S13 and S15) after the exhaustion of excess fuel is probably due to an autocatalytic path involving deprotonated **3** itself as the catalyst. Such complex mechanism has been studied in detail in the simpler case of calix[4]arene **2**.^9c^ In that case, it was shown that the fact that the rate of the autocatalytic process does not slow down on decreasing the substrate (fuel) concentration, with the consequent presence of a cusp just before the final plateau, can be explained considering the autocatalytic path to be kinetically zero order with respect to the substrate itself.^9c,22^ The presence of minute amounts of cationic impurities in dichloromethane was shown to be likely responsible for such bizarre kinetic behavior.^9c^

## conclusions

In this report, we have shown that the host–guest interaction between calix[6]arene **3** and *N*-methylisoquinolinium triflate **4** can be dissipatively driven by fuel acid **1d**. The addition of the fuel into a solution containing the complex **3**•**4** causes the temporary release of guest **4** by the host into the bulk. The former is re-uptaken by the latter once the fuel is exhausted. The amount of released guest and the duration of the unloaded state can be controlled at will by a fine modulation of the quantity of added fuel. Thus, the fully abiotic host–guest couple (**3**•**4**) described above is capable of operating under dissipative conditions (out of equilibrium, as long as the fuel is present), which are typical, operative conditions of systems with life-like properties.

A possible development of the present work could be the design of systems where the dissipative complex dissociation gives rise to a secondary effect. The temporal control of catalytic activity may be, for example, obtained in case the guest is a catalyst of a given reaction.^23^

## Supporting Information Available

Experimental section, ^1^H NMR spectra of **4**, ^1^H NMR titration experiments, variable temperature ^1^H NMR spectra of **3** and **3**•**4**, kinetic experiments, identification of peroxide **5** (PDF)
