# Supplementary material for: Temporal Control of the Host–Guest Properties of a Calix[6]arene Receptor by the Use of a Chemical Fuel
Source: J Org Chem. 2022 Feb 23;87(5):3623–9. doi: 10.1021/acs.joc.2c00050 (PMC8902750; doi:10.1021/acs.joc.2c00050)
Supplement: Supplementary file 1 — jo2c00050_si_001.pdf [file jo2c00050_si_001.pdf]

Supporting Information for:

Temporal Control of the Host-Guest Properties of a Calix[6]arene Receptor by Use of a Chemical Fuel

Francesco Rispoli,[a] Emanuele Spatola,[b] Daniele Del Giudice,[b] Roberta Cacciapaglia,[b] Alessandro Casnati[a], Laura Baldini,[a,] and Stefano Di Stefano[b,]**

[a]Dipartimento di Scienze Chimiche, della Vita e della Sostenibilità Ambientale Università degli Studi di Parma Parco Area delle Scienze 17/A, 43124 Parma, Italy. E-Mail: laura.baldini@unipr.it

[b]Dipartimento di Chimica Università di Roma La Sapienza and ISB-CNR Sede Secondaria di Roma - Meccanismi di Reazione, P.le A. Moro 5, I-00185 Roma, Italy. E-mail: stefano.distefano@uniroma1.it

Table of Contents **page**

1. **Experimental Section** **S2**
   - 1.1 Instruments and Methods
   - 1.2 Experimental Methods
     - 1.2.1 NMR titration of **3** with TFA
     - 1.2.2 NMR titration of **3** with **4**
     - 1.2.3 NMR decarboxylation experiment of fuel **1d** in the presence of 2.0 mM calix[6]arene **3**
     - 1.2.4 NMR decarboxylation experiment of fuel 1d in the presence of 2.0 mM calix[6]arene **3** and 2 mM N-methylisoquinolinium trifluoromethanesulfonate **4**

2. **^{1}H NMR spectra** **S3**
   - 2.1 ^{1}H NMR spectrum of 2.00 mM N-methylisoquinolinium trifluoromethanesulfonate **4**
   - 2.2 ^{1}H NMR titration of **3** with 4
   - 2.3 ^{1}H NMR spectrum of **3** at varying temperature
   - 2.4 ^{1}H NMR spectrum of complex **3•4** at varying temperature
   - 2.5 ^{1}H NMR titration of **3** with TFA
   - 2.6 ^{1}H NMR titration of **3**H^+ with p anisidine **6**
   - 2.7 ^{1}H NMR monitoring of the effect of protonation on the binding ability of **3**
   - 2.8 ^{1}H NMR monitoring of the reaction between 2.00 mM **3** and 6.00 mM **1d**
   - 2.9 ^{1}H NMR monitoring of the reaction between 2.00 mM **3** and 6.00 mM **1d** in the presence of 2.00 mM **4**
   - 2.10 ^{1}H NMR onitoring of the reaction between 2.00 mM **3** and 10.5 mM **1d** in the presence of 2.00 mM **4**
   - 2.11 ^{1}H NMR Monitoring of the Reaction between 2.00 mM **3** and 18.2 mM **1d** in the presence of 2.00 mM **4**

3. **Identification of peroxide 5** **S14**

1. Experimental Section

1.1 Instruments and methods

^1H NMR spectra were recorded on a Bruker300 MHz spectrometer. The spectra were internally referenced to the residual proton signal of the solvent at 5.32 ppm (CD_2Cl_2). Unless otherwise stated, all the experiments were carried out at 25°C. GC-MS analysis was performed with a gas chromatograph equipped with a silica capillary column (30 m x 0.2 mm x 25 µm) OV5 (95% methylsilicone, 5% phenylsilicone) coupled with a mass detector (EI, 70eV).

1.2 Experimental Methods

1.2.1 NMR titration of 3 with TFA

Calix[6]arene **3** (1.12 mg) was weighed into an NMR tube and diluted with 580 µL of CD_2Cl_2 to give a 2.00 mM solution. At this point, subsequent aliquots of 5.0 µL of a 0.060 M stock solution of TFA were added up to 8 mol equiv. NMR spectra of the solution were recorded at each titrant addition.

1.2.2 NMR titration of 3 with 4

Calix[6]arene **3** (1.12 mg) was weighed into an NMR tube and diluted with 600 µL of CD_2Cl_2 to give a **3** 2.00 mM solution. At this point, subsequent aliquots of 5.0 µL of a 0.24 M **4** and 2.00 mM **3** stock solution were added up to 54 mol equiv. NMR spectra of the solution were recorded at each titrant addition.

1.2.3 NMR decarboxylation experiment of fuel 1d in the presence of 2.0 mM calix[6]arene 3

1.12 mg of calix[6]arene **3** were weighed into an NMR tube and diluted with CD_2Cl_2 giving a solution 2.00 mM. The t = 0 ^1H NMR spectrum was recorded and then an aliquot of a stock solution of **1d** was added so that the final volume of the solution was 600 µL. Thus, concentration of calix[6]arene **3** was 2.00 mM, and that of fuel **1d** was 6.00 mM.

1.2.4 NMR decarboxylation experiment of fuel 1d in the presence of 2.0 mM calix[6]arene 3 and 2 mM *N*-methylisoquinolinium trifluoromethanesulfonate 4

In a typical NMR decarboxylation experiment, 1.12 mg of calix[6]arene **3** were weighed into an NMR tube and diluted with CD_2Cl_2 giving a solution 2.00 mM solution. Then 50 µL of a 24.0 mM stock solution of **4** were added. The t = 0 ^1H NMR spectrum was recorded and then an aliquot of a stock solution of **1d** was added so that the final volume of the solution was 600 µL. Thus, concentrations of calix[6]arene **3** and *N*-methylisoquinolinium trifluoromethanesulfonate **4** were 2.00 mM, while that of fuel **1d** was 6.00 mM or higher for the experiments with fuel excess.

2. ^{1}H NMR spectra

2.1 ^{1}H NMR spectrum of 2.00 mM *N*-methylisoquinolinium trifluoromethanesulfonate 4.

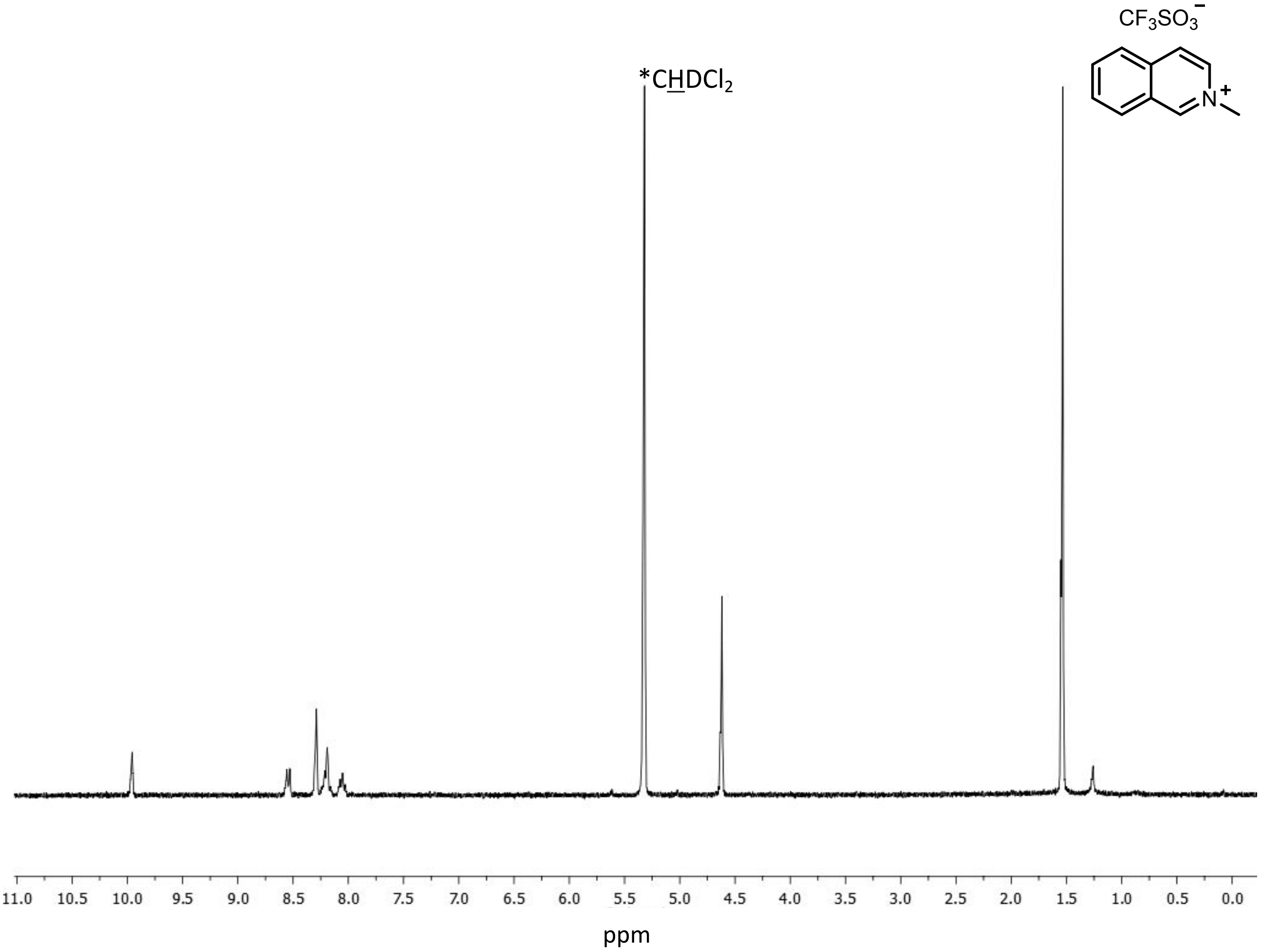

Figure S1. ^{1}H NMR spectrum (CD_2Cl_2, 300 MHz, 25 °C) of 2.0 mM *N*-methylisoquinolinium trifluoromethanesulfonate **4**. δ (ppm): 9.95 (s, 1H, Ar-H); 8.54 (d, 1H, Ar-H); 8.34-8.27 (m, 2H, Ar-H); 8.26-8.14 (m, 2H, Ar-H); 8.05 (t, 1H, Ar-H); 4.61 (s, 3H, CH_3).

2.2 ^{1}H NMR titration of 3 with 4.

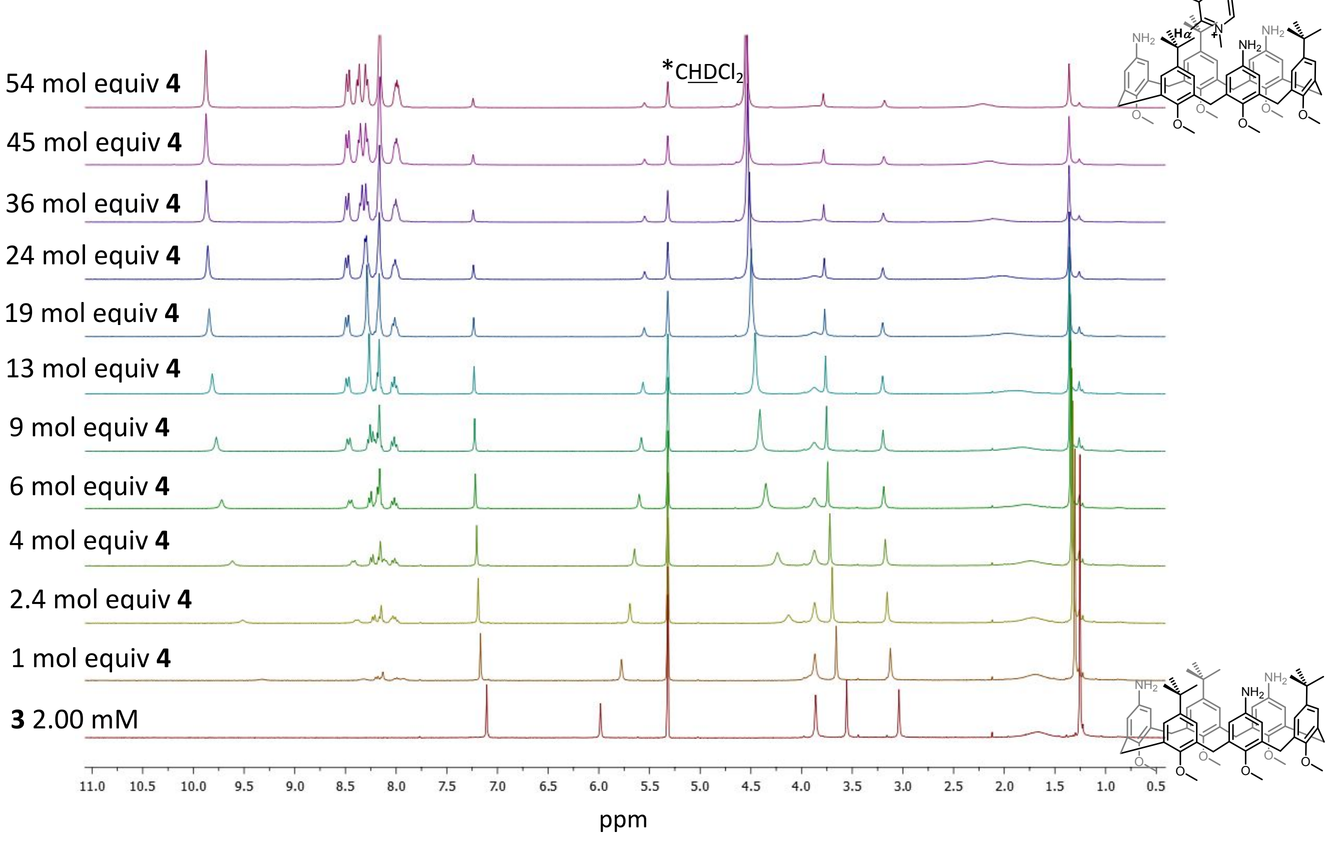

Figure. S2. ^{1}H NMR (CD_2Cl_2, 300 MHz, 25 °C) titration of calix[6]arene **3** with **4**. From bottom to top: spectrum of 2.00 mM **3**, and spectra registered after the addition of increasing amounts of **4**, as reported.

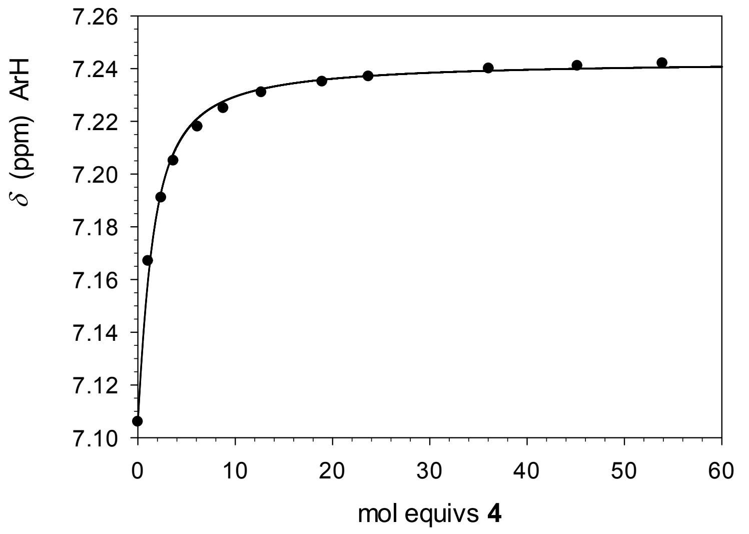

Figure S3. ^{1}H-NMR (CD_2Cl_2, 300 MHz, 25 °C) titration of a 2.0 mM **3** solution with **4**. The chemical shift of the signal of the aromatic proton of *t*-Bu substituted aromatic rings is plotted *vs*. added molar equivs of **4**. The curve is a 1:1 binding isotherm calculated with best fit values K_{ass} = 500 M^{-1} , and $\delta_{3\cdot 4}$ = 7.24 ppm.

2.3 ^{1}H NMR spectrum of 3 at varying temperature

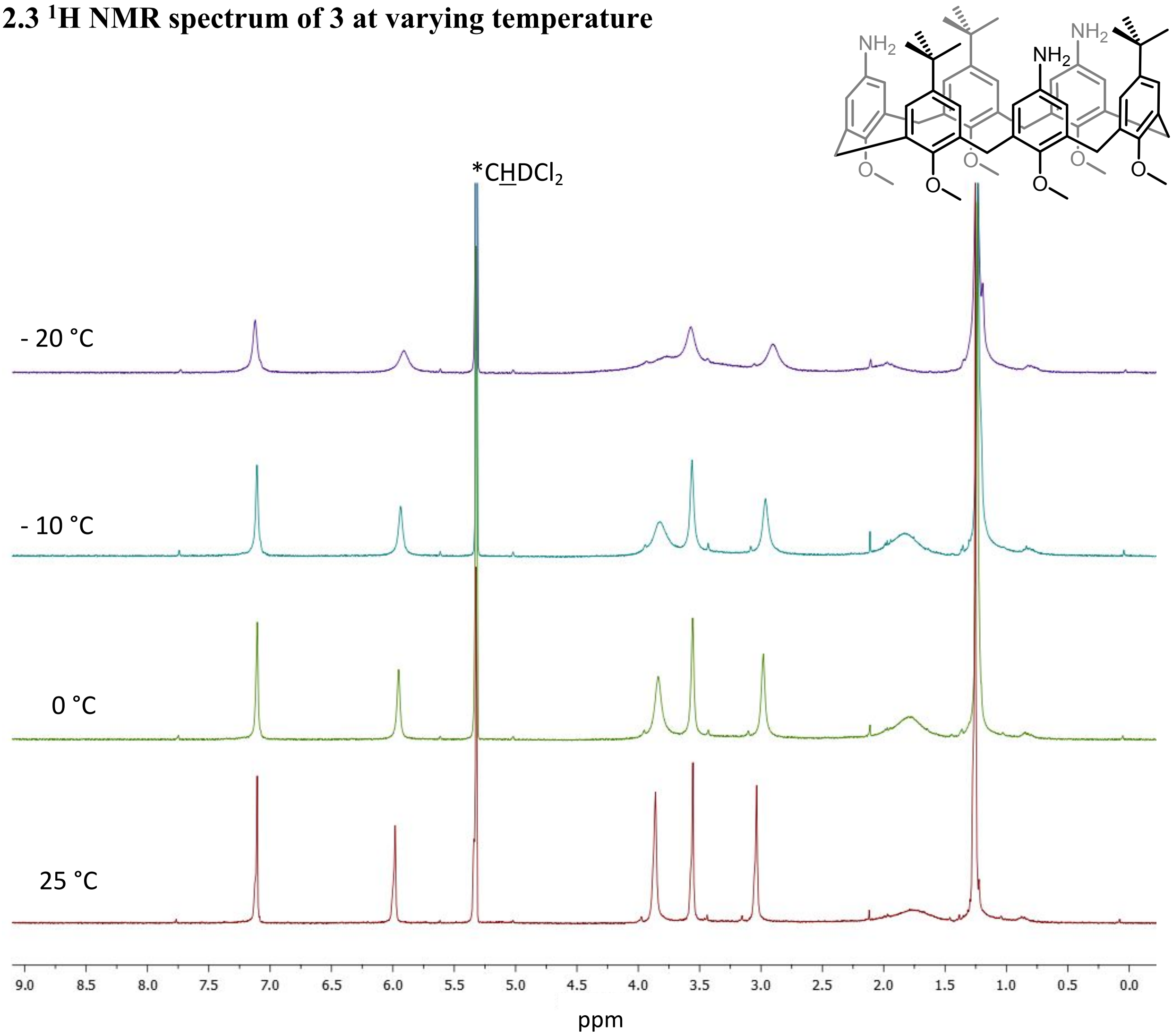

Figure S4. ^{1}H NMR spectra (CD_2Cl_2, 300 MHz) of 2.00 mM **3** at varying temperature**.**

2.4 ^{1}H NMR spectrum of complex 3•4 at varying temperature

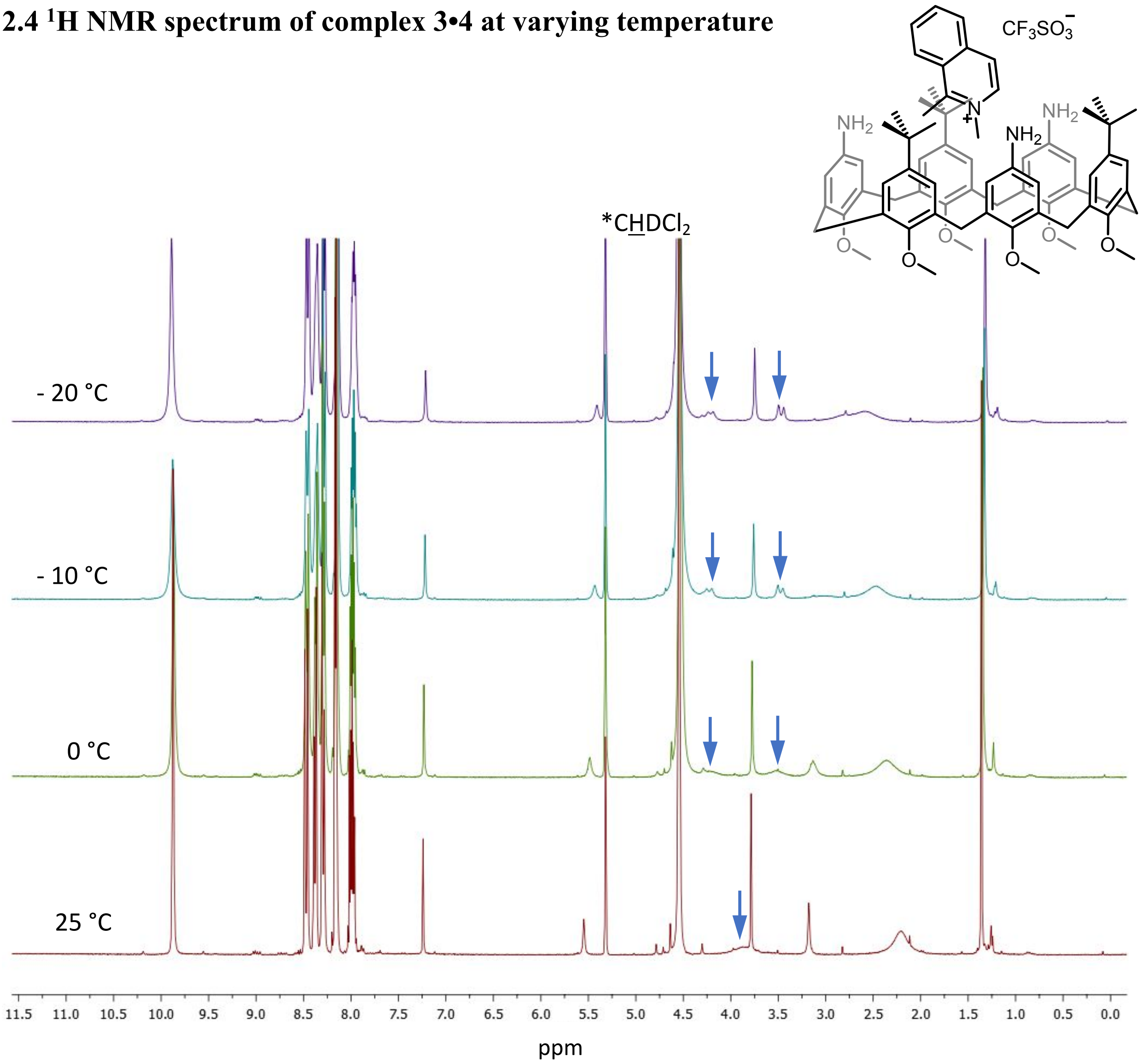

Figure S5. ^{1}H NMR spectra (CD_2Cl_2, 300 MHz) of 2.00 mM **3** + 100 mM **4** (approximately 98% complex **3•4** at varying temperature**.** Blue arrows show the splitting of the signals belonging to the methylene bridges upon cooling.

2.5 ^{1}H NMR titration of 3 with TFA.

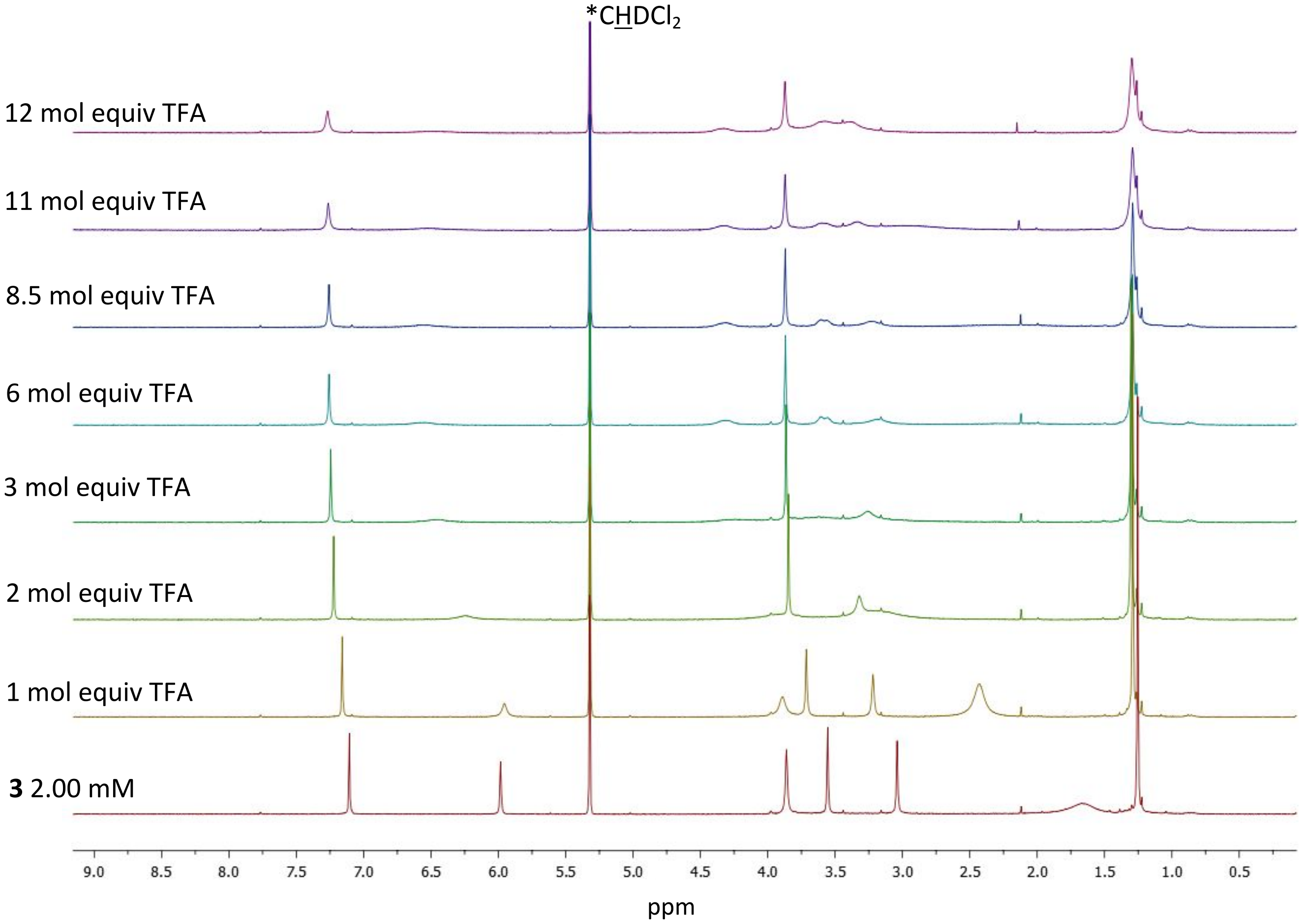

Figure S6. ^{1}H NMR (CD_2Cl_2, 300 MHz, 25 °C) titration of calix[6]arene **3** with TFA. From bottom to top: spectrum of 2.00 mM **3**, and spectra registered after the addition of increasing amounts of TFA, as reported.

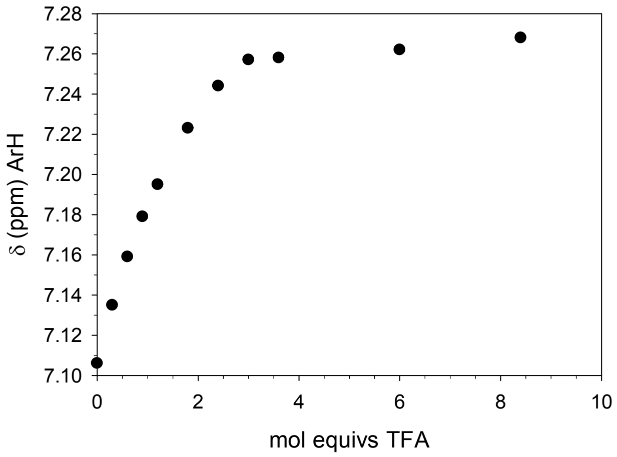

Figure S7. Plot of the chemical shift of the *t*-Bu substituted aromatic rings signal on increasing the amount of TFA.

2.6 ^{1}H NMR titration of $3H^{+}$ with *p*-anisidine 6.

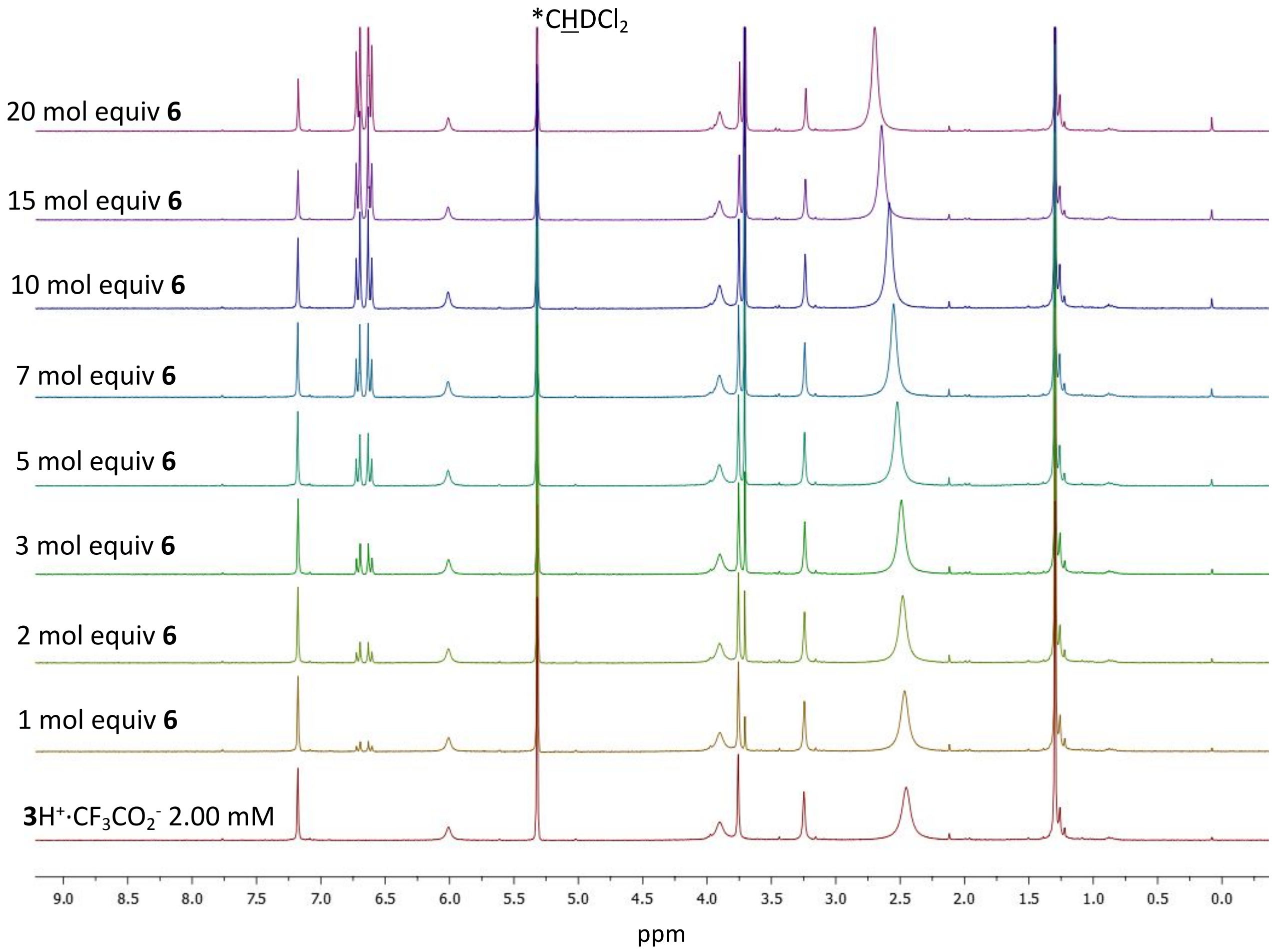

Figure S8. ^{1}H NMR (CD_2Cl_2, 300 MHz, 25 °C) titration of **3**$H^{+}\cdot CF_3CO_2^{-}$ (2.00 mM **3** + 2.00 mM TFA) with *p*-anisidine **6**. From bottom to top: spectrum of 2.00 mM **3**$H^{+}\cdot CF_3CO_2^{-}$, and spectra registered after the addition of increasing amounts of *p*-anisidine **6**.

2.7 ^{1}H NMR monitoring of the effect of protonation on the binding ability of 3.

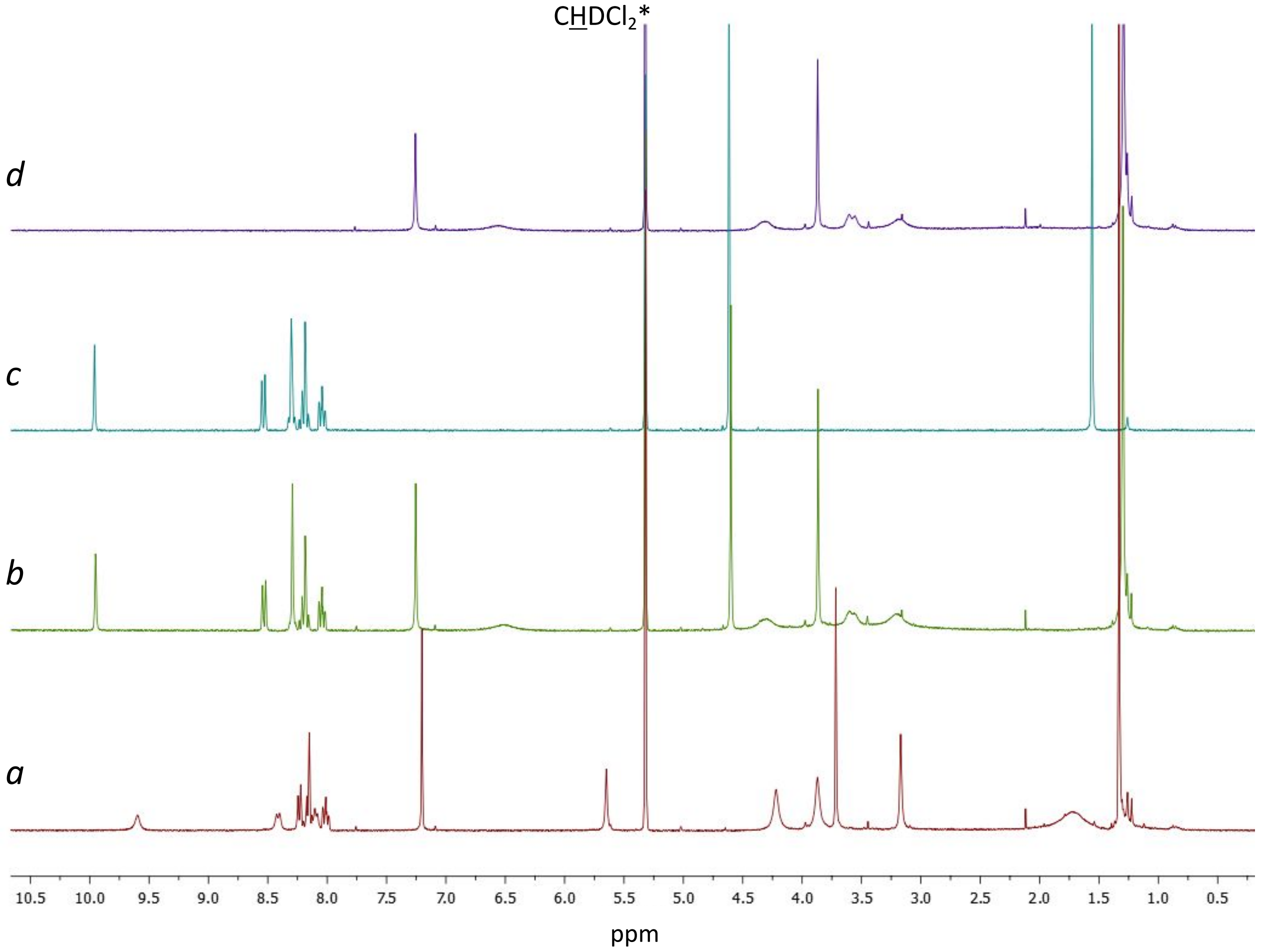

Figure S9. ^{1}H NMR monitoring (CD_2Cl_2, 300 MHz, 25 °C) of the effect of protonation on the binding ability of **3** towards isoquinolinium triflate **4**. 2.00 mM **3** + 6.00 mM **4** (trace *a*); 2.00 mM **3** + 6.00 mM **4** + 6.00 mM TFA (trace *b*); 6.00 mM **4** (trace *c*); 2.00 mM **3** + 6.00 mM TFA (trace *d*).

2.8 ^{1}H NMR monitoring of the reaction between 2.00 mM 3 and 6.00 mM 1d.

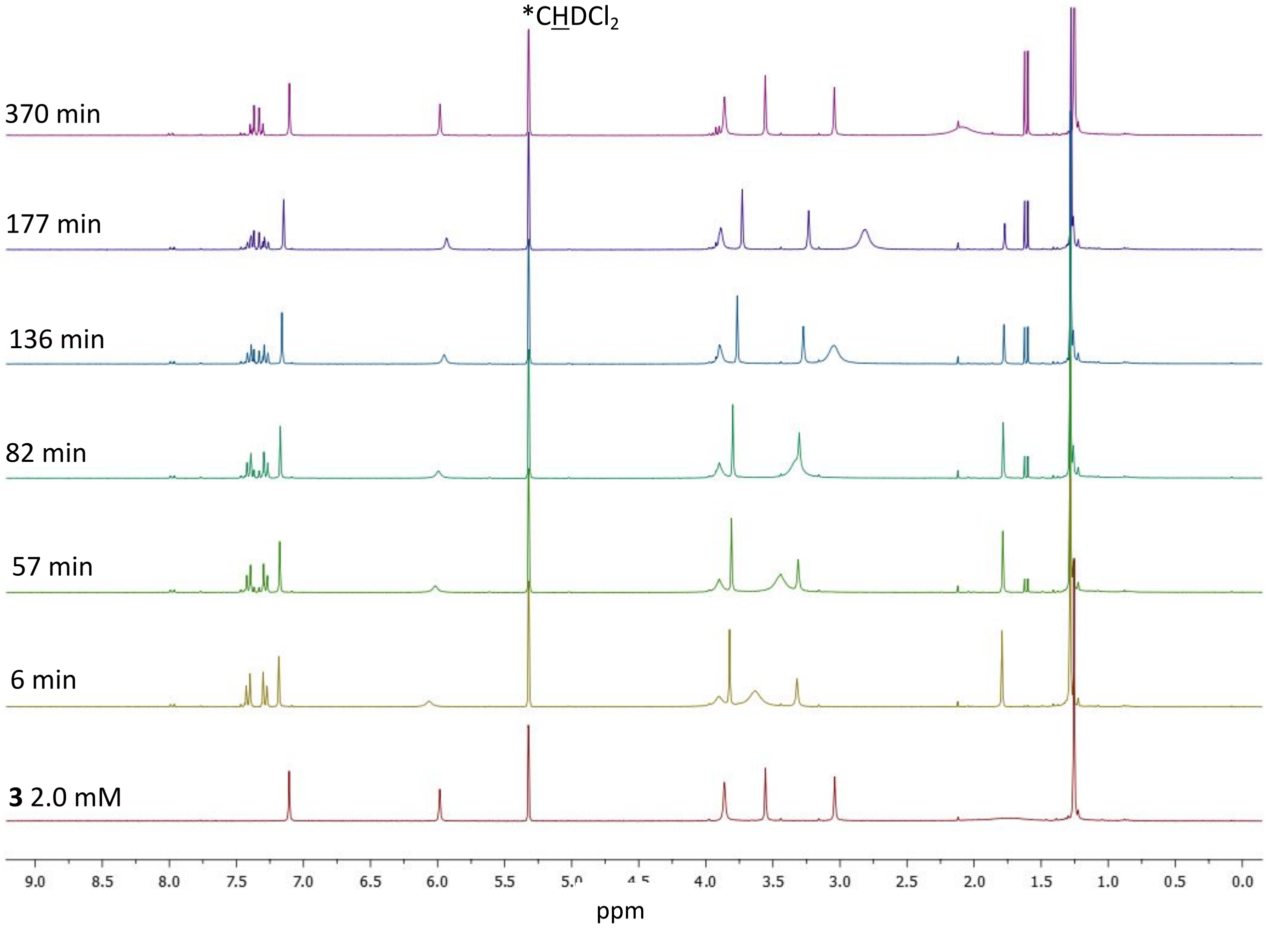

Figure S10. ^{1}H NMR monitoring (CD_2Cl_2, 300 MHz, 25 °C) of the decarboxylation of 6.00 mM **1d** catalysed by 2.00 mM **3**. The bottom trace is relative to 2.00 mM **3**, before the addition of **1d**. Following spectra were recorded after the addition of acid **1d**, at the given reaction time, upward increasing.

2.9 ^{1}H NMR monitoring of the reaction between 2.00 mM 3 and 6.00 mM 1d in the presence of 2.00 mM 4.

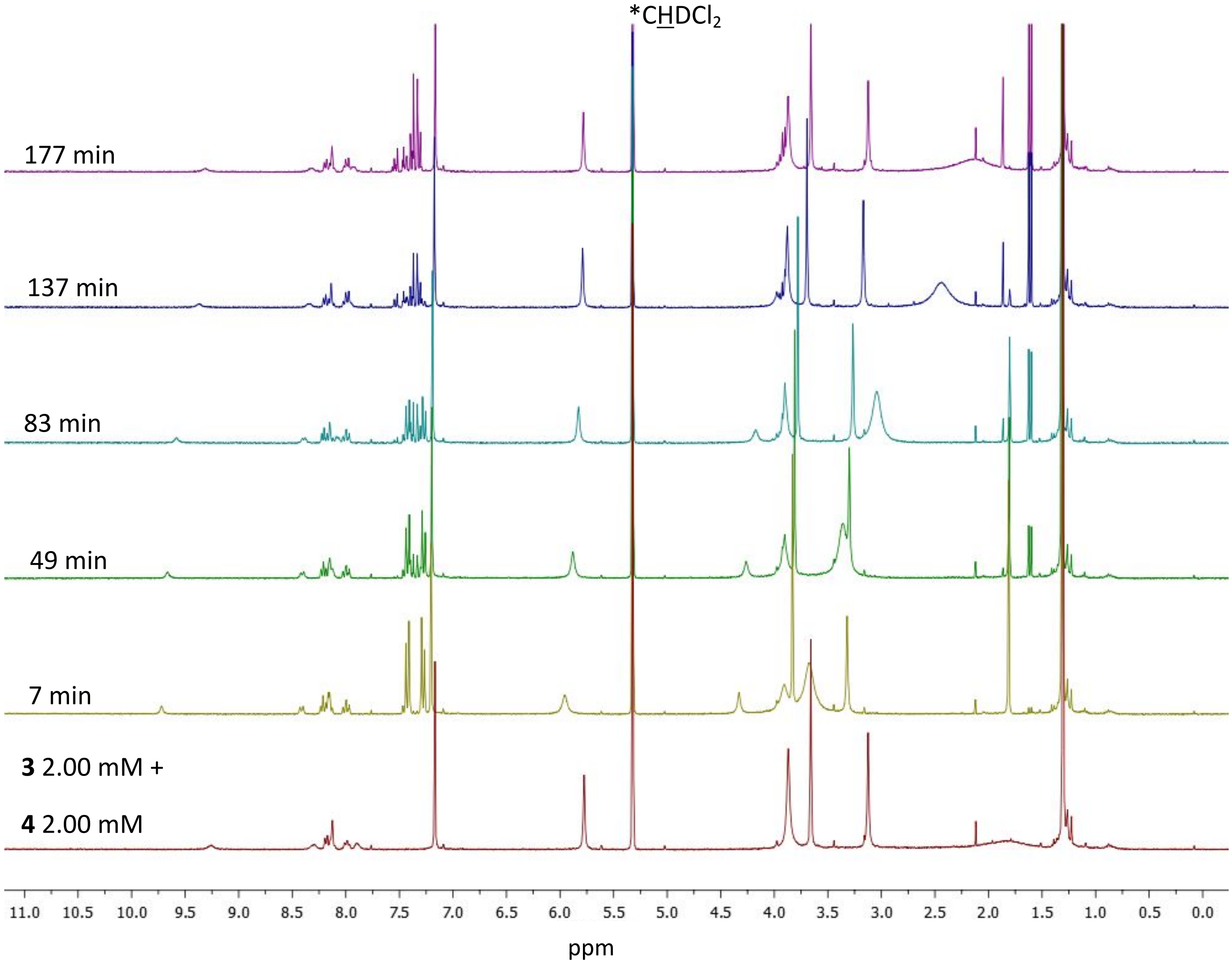

Figure S11. ^{1}H NMR monitoring (CD_2Cl_2, 300 MHz, 25 °C) of the decarboxylation of 6.00 mM **1d** catalysed by 2.00 mM **3** in the presence of 2.00 mM **4**. The bottom trace is relative to 2.00 mM **3** + 2.00 mM **4**, before addition of **1d**. Following spectra were recorded after the addition of acid **1d**, at the given reaction time, upward increasing.

2.10 ^{1}H NMR monitoring of the reaction between 2.00 mM 3 and 10.5 mM 1d in the presence of 2.00 mM 4.

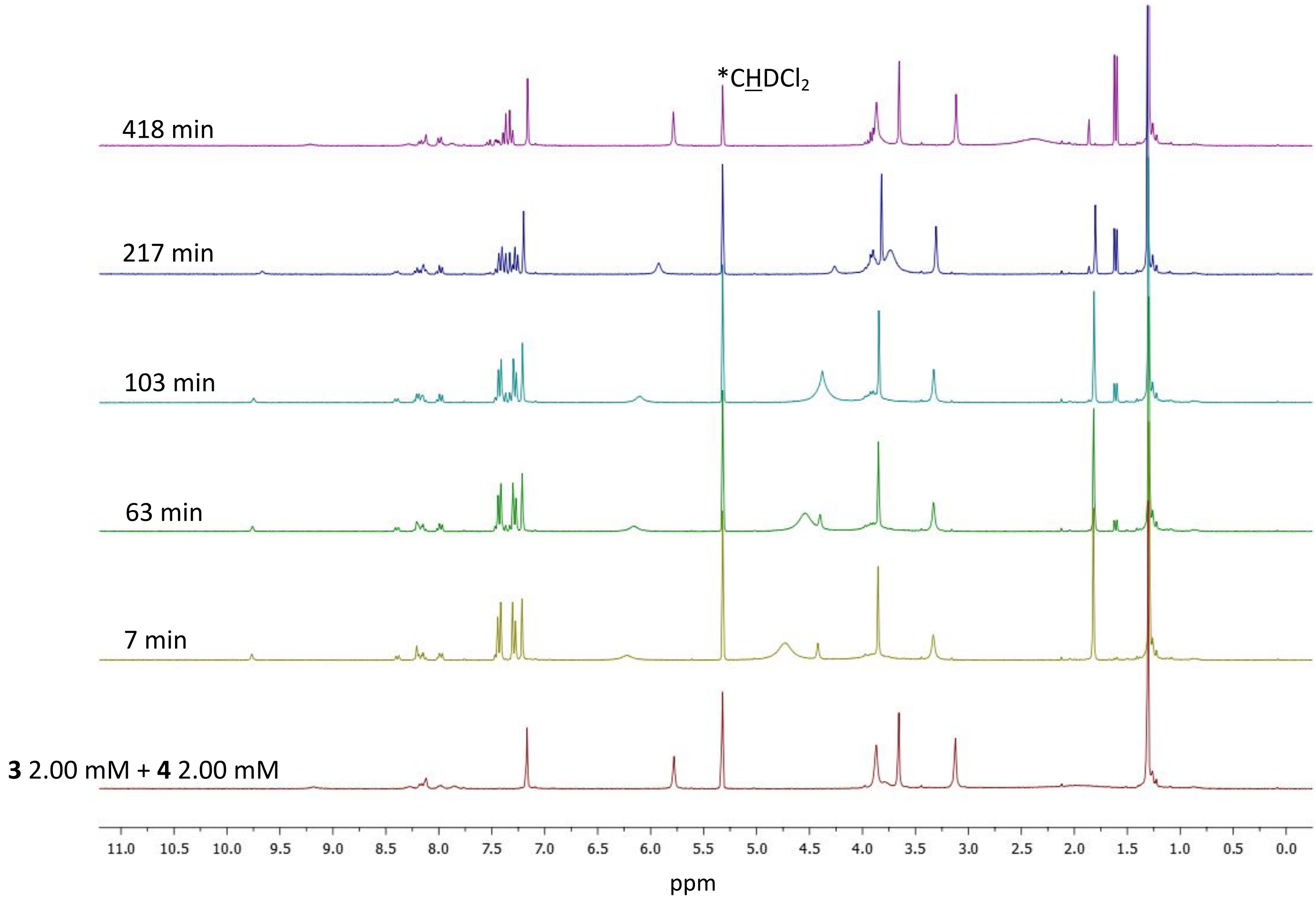

Figure S12. ^{1}H NMR monitoring (CD_2Cl_2, 300 MHz, 25 °C) of the decarboxylation of 10.5 mM **1d** catalysed by 2.0 mM **3** in the presence of 2.00 mM. The bottom trace is relative to 2.00 mM **3** + 2.00 mM **4**, before the addition of **1d**. Following spectra were recorded after the addition of acid **1d**, at the given reaction time, upward increasing.

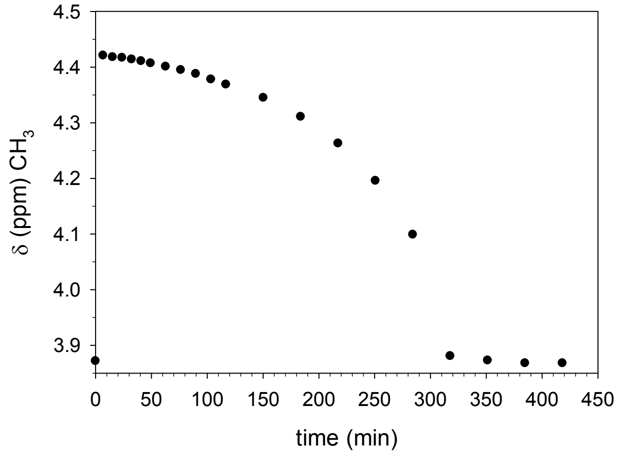

Figure S13. ^{1}H NMR monitoring (CD_2Cl_2, 300 MHz, 25 °C) of the decarboxylation of 10.5 mM **1d** catalysed by 2.00 mM **3** in the presence of 2.00 mM **4**. Data points are obtained following the chemical shift of the methyl of **4** over time.

2.11. ¹H NMR Monitoring of the Reaction between 2.00 mM 3 and 18.2 mM 1d in the presence of 2.00 mM 4.

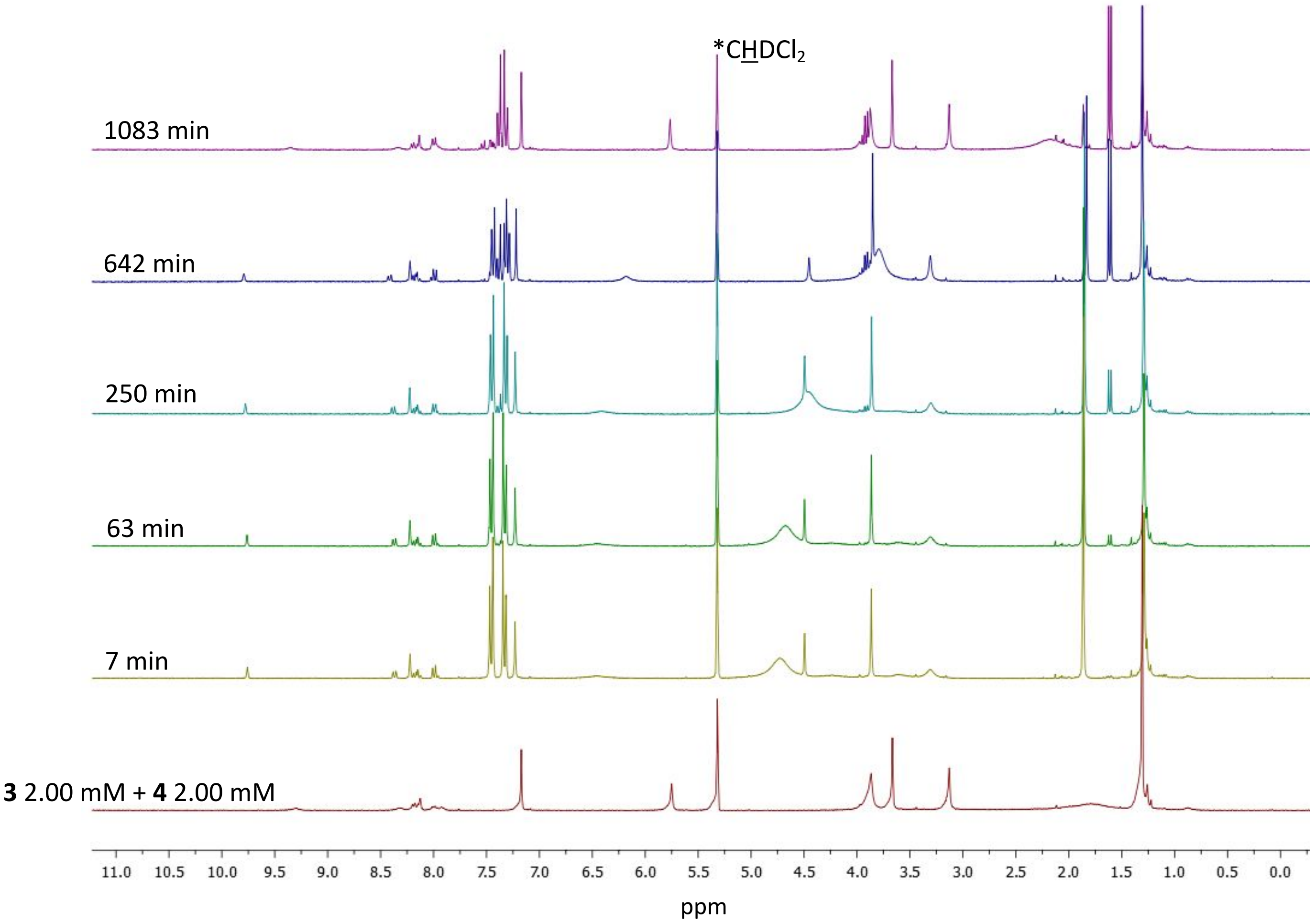

Figure S14. 1H NMR monitoring (CD_2Cl_2, 300 MHz, 25 °C)of the decarboxylation of 18.2 mM **1d** catalysed by 2.00 mM **3** in the presence of 2.00 mM **4**. The bottom trace is relative to 2.00 mM **3** + 2.00 mM **4**, before the addition of **1d**. Following spectra were recorded after the addition of acid **1d**, at the given reaction time, upward increasing.

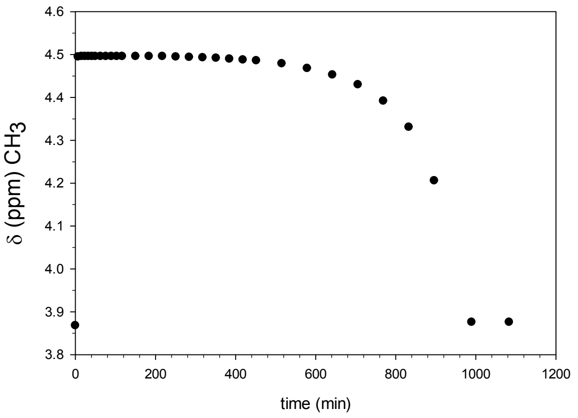

Figure S15. ¹H NMR monitoring (CD_2Cl_2, 300 MHz, 25 °C) of the decarboxylation of 18.2 mM **1d** catalysed by 2.00 mM **3** in the presence of 2.00 mM **4**. Data points are obtained following the chemical shift of the methyl of **4** over time.

3. Identification of peroxide 5

The identification of the waste co-product **5** was quite puzzling. First of all, the product was eluted through a Pasteur pipette by column chromatography carried out on the solution of the ^{1}H NMR experiment described in Figure S11. The figure below shows the ^{1}H NMR of the fraction containing tiny amounts of **5**.

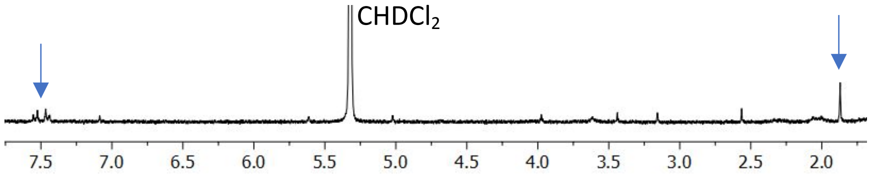

The AB system centered at 7.5 ppm and the singlet at 1.80 ppm belong to compound **5**. The same signals are easily found for example in the top spectrum of Figure S11. This spectrum compares well with the spectrum simulated for the peroxide **5**.

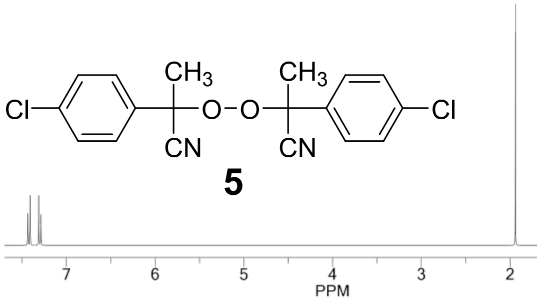

When the above CD_2Cl_2 solution was subjected to MS analysis, we found the same mass spectra that is obtained for *p*-chloroacetophenone. This fact can be justified by the loss of cyanogen in the injector of the mass spectrometer according to the mechanism illustrated below.

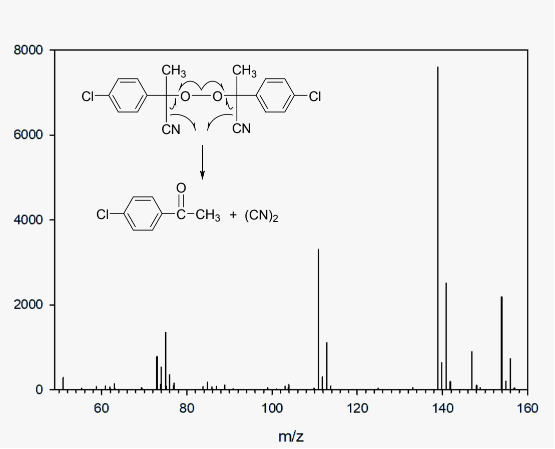

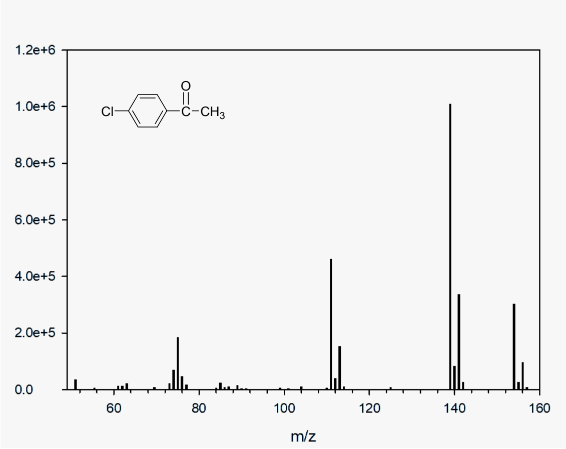

However, the structure of the waste co-product is certainly not that of *p*-chloroacetophenone whose methyl group signal is found at 2.56 ppm (^{1}H NMR in CD_2Cl_2). Oxidation to a radical species of a carbanion similar to that reported in Figure 7 (see main text) by aromatic quaternary salts and subsequent capture of O_2, is well documented (see ref 9b in the main text). Furthermore, structure **S1** deriving by the direct coupling of two just formed carbon radicals can be excluded since it is known (Kojitani, T. *J. Org. Chem.* **1975**, *40*, 3540–

3544) that the methyl groups of the two possible diasteroisomers have slightly different absorption at ^{1}H NMR (we should observe two singlets around 1.8 ppm and not one only, see also ref 17 of the main text).

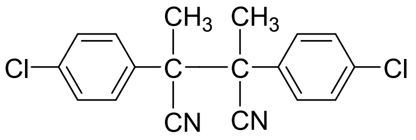

S1

The above considerations, together with the findings that the contribution of the collateral pathway definitely decreases when: i) exclusion of O_2 from the solution was attempted through freeze-pump-thaw cycles operated on the NMR tube (passing from 25% to 15%), and ii) higher excesses of fuel **1d** were employed, strongly points to the peroxide **5** as the structure of the waste co-product. Again, it has to be stressed that the collateral pathway doesn't interfere on the releasing-reloading cycles.
